# G Protein-Coupled Estrogen Receptor in Immune Cells and Its Role in Immune-Related Diseases

**DOI:** 10.3389/fendo.2020.579420

**Published:** 2020-10-02

**Authors:** George Notas, Marilena Kampa, Elias Castanas

**Affiliations:** Laboratory of Experimental Endocrinology, School of Medicine, University of Crete, Heraklion, Greece

**Keywords:** GPER1, estrogen receptor, immune cells, inflammation, TLR4

## Abstract

G protein-coupled estrogen receptor 1 (GPER1), is a functional estrogen receptor involved in estrogen related actions on several systems including processes of the nervous, reproductive, metabolic, cardiovascular, and immune system. Regarding the latter, GPER is expressed in peripheral B and T lymphocytes as well as in monocytes, eosinophils, and neutrophils. Several studies have implicated GPER in immune-mediated diseases like multiple sclerosis, Parkinson’s disease, and atherosclerosis-related inflammation, while a recent report suggests that its deletion could be responsible for a form of familial immunodeficiency. It has also been suggested that it is a key regulator of immune-mediated events in breast, pancreatic, prostate, and hepatocellular cancer as well as in melanoma. GPER has been also reported to interact with classic ER-alpha or its splice variants in order to modify immune functions. This review aims to present current knowledge relating GPER to immune functions, the cellular and signaling pathways involved, as well as the potential clinical implications of GPER modulation in immune-related diseases.

## Introduction

Our perception of the mechanisms involved in estrogen (patho)physiological effects has progressed significantly in the last fifteen years, with the discovery of G protein-coupled estrogen receptor 1 (GPER1, previously known as G protein-coupled receptor 30 or GPR30). GPER1 is a seven transmembrane-domain G protein-coupled receptor (GPCR) that in 2005 was reported independently by two research groups to bind 17β-estradiol (E2) with high affinity and to induce unique and specific signaling, upon its activation by this ligand (1, 2). GPER1 was at the time the answer for the rapid estrogen actions pointing out the need for a paradigm shift in the field. However, parallel reports for membrane anchoring of classic ERα and ERβ *via* palmitoylation, tethered actions, and role of specific ER splice variants, further added to the complexity of rapid, extranuclear steroid signaling (3). Hundreds of studies further explored the role of GPER1 in cellular physiology and the regulation of rapid steroid actions. As a result, the role of GPER1 in estrogen actions in several systems is now well accepted, even though not fully elucidated yet.

Most of the GPER1 actions are mediated through the rapid activation of G proteins, Adenylyl cyclase/PKA, tyrosine kinases, the membrane-associated guanylate kinase (MAGUK) family of PDZ domain proteins, MAP kinases, and PI3K (4–6). Moreover, several lines of evidence also suggest that, upon GPER1 activation, specific actions on gene expression can be modified (7). Apart from estradiol, a variety of drugs, phytoestrogens, and xenoestrogens were found to exert actions *via* GPER1, while potent synthetic agonists and antagonists have been synthesized and utilized to understand GPER1-dependent actions and specific estradiol biologic effects (8). Multiple research groups have identified specific GPER1 actions in distinct cell types, organs, and systems. The research produced in this field in the last 15 years is now reaching maturity and GPER1 targeting is now also studied as a novel therapeutic approach in cancer, cerebrovascular, metabolic, and neurodegenerative diseases (9–11). Indeed, GPER1 has been found to regulate estrogenic effects on specific immune functions, not only in humans but also in various other species (12–16). Therefore, research regarding the role of this membrane receptor in diseases characterized by sexual dimorphism, like atherosclerosis, some types of cancer, and several autoimmune conditions, could provide further insight into the pathophysiology of these diseases and create opportunities for novel therapies.

The role of GPER1 in the immune system is another field of potential GPER1 actions. The immunomodulatory effect of GPER1 has also been implicated in cancer immune tolerance, although data for potential therapeutic implications in this field are limited (17, 18). In this review, we will analyze the current knowledge regarding the expression of GPER1 in the immune system and will review the diseases, or disease-models, where this receptor might play an important pathophysiological role. Additionally, the signaling mechanisms involved and the interaction of GPER1 with critical molecules regulating major immune functions will also be discussed.

## Expression and Function of GPER1 by Cells of the Immune System

GPER1 mRNA is expressed in CD34+, CD38+ hemopoietic cells, and mature cells of the immune system. Its appearance in the early stages of immune cell development suggests its role in their maturation and function (19, 20). The functional role of GPER1 in each population of immune cells has been explored in several published works. However, the depth of our knowledge is still limited and several controversies have arisen from conflicting results, as presented in **Table 1**. Below, we summarize the findings of GPER1 detection and actions in specific immune cell populations:

**Table 1:** Actions attributed to GPER1 in each cell type of the immune system.

Cell type	Action	Reference
T-lymphocytes	• Induces thymic atrophy (controversial)	(21–25)
	• Induces IL-10 in CD4+ T cells	(26, 27)
	• Enhances CD4+ T cells Foxp3 expression and Foxp3 positive T-cells	(28)
	• Increases T-cell proliferation (fish)	(29, 30)
B-lymphocytes	• Decreases activation‐induced B cell proliferation • Increases IgG (memory)	(29)
	• Inhibits proliferation (fish)	(30)
	• Enhances natural antibody production (mice)	(30).
Monocytes/ macrophages	• Decreases TLR4 expression, • Blocks the inflammatory response to LPS and expression of PGE2, IL-6, and TNFα	(31)
	• Mediates E2 anti-inflammatory action on LPS activated human monocytes and *in vitro* differentiated macrophages *via* interaction with ERα36 and NFκB and blocks IL-6 and TNFα release	(32)
	• Inhibits hepatocarcinogenesis in the DEN induced HCC model by inhibiting IL-6 expression by Kupffer cells	(33)
Eosinophils	• Increases CCL11 induced chemotaxis • Blocks caspase-3 dependent spontaneous apoptosis in resting eosinophils • Increases apoptosis in IL-5 stimulated eosinophils.	(34)
	• Suppresses airway inflammation (mouse asthma model)	(35)
Neutrophils	• Increases IL1b, CXCL8, and COX2 expression • Enhances respiratory burst • Increases life span	(36)
	• Has anti-inflammatory effects in the equivalent of human neutrophils in fish	(13, 37, 38)

### Lymphocytes

GPER1 is expressed in bone marrow B lineage CD19+ IgM- cells (pro- and pre-B cells), in peripheral B cells, and in circulating T cells (19, 21, 29). Peripheral T and B cells express GPER1, showing a distinct subcellular distribution, different from the classical ERα and ERβ. These cells also exhibit membrane binding sites for estrogen that are attributed to GPER1 and membrane-bound forms of the classic estrogen receptors, which display significant ligand-dependent internalization and recycling (29).

#### T Lymphocytes

In T-cells, GPER1 expression has been specifically reported in CD4+ CD44^lo^CD62L^hi^Foxp3^-^ naive T‐cell and CD4+ Foxp3+ T‐reg cells (28). GPER1 has been found to affect cytokine expression, lineage progression, and proliferation. Although the mechanisms involved are not fully elucidated, early GPER1 expression in adaptive immunity-related cells could affect their maturation. Early reports disclosed that estrogen mediates thymic atrophy *via* the classic estrogen receptor ERα (39). Interestingly, after the discovery of GPER1, it was found that its loss in mice leads to partial resistance to E2-induced thymic atrophy *via* thymocyte/naive T-cell apoptosis. In contrast, in wild-type mice, the GPER1 agonist G-1 effects on thymic atrophy were equivalent to that of estrogen (19, 21). This finding, however, could not be reproduced in three other early studies (22, 23), using a different KO mouse strain.

The role of GPER1 in the induction of IL-10 expression by CD4+ T cells and especially in the T helper 17 (Th17) subpopulation, has been studied more thoroughly; GPER1 specific agonist G1 increases IL-10 expression in these cells, activating the ERK kinase pathway, a well-established signaling pathway of this receptor (26, 27). Increased IL-10 expression was also observed *in vivo*, in splenocytes isolated from G1-treated male mice (26). G-1 also enhances Foxp3 expression in CD4+ T cells and increases the number of Foxp3+ T-cells, when they are polarized *in vitro* toward the Th17 lineage (28). In the same study, it is reported that G1 also induces small increases in the expression of PD-1 and CTLA-4. It is to note that prior investigations have attributed this estrogen-elicited action to an effect of the classical estrogen receptors (40, 41).

Finally, although estrogen has been reported to decrease activation‐induced T cell proliferation, E2‐BSA, acting exclusively on membrane estrogen receptors, enhanced cell growth. In addition, in fish, estrogen increases the proliferation of T-cells specifically *via* GPER1 (29, 30).

#### B-Lymphocytes

Similarly to T-cells, activation‐induced B cell proliferation is decreased by estrogen, while membrane-only acting estrogen enhances it. The effect of membrane acting estrogen, potentially *via* GPER1, also increases IgG production in mice, but only when immune memory has been established (29). This suggests that GPER1 may affect the function of memory B-lymphocytes or plasmacytes, a finding that should be further studied, since it may explain recorded gender-specific differences in adaptive immunity. In fish, however, GPER1 mediates estrogen-dependent inhibition of B-cell proliferation GPER1 (30), while both estrogen and G1 also raise natural antibody production in mice *via* GPER1 (30).

### Monocytes/Macrophages

Several research groups have reported that GPER1 is expressed in monocytic cell lines, CD14+ monocytes, in *in vitro* differentiated macrophages and in tissue-resident macrophages (19, 31, 32, 42).

It has been repeatedly shown that estrogen can inhibit monocyte/macrophages activation and this was attributed to the classical estrogen receptors (43). This estrogenic action could be crucial for diseases that display sexual dimorphism, like atherogenesis, asthma, and some types of cancer. It was later found that GPER1 mediates the anti-inflammatory effect of estrogen in the monocyte/macrophages population through multiple mechanisms. Both 17beta-estradiol and G1 decrease TLR4 expression in RAW 264.7 cells and primary mouse peritoneal macrophages and this effect is abolished in GPER1 knockdown cells. Treatment of RAW 264.7 cells with G1 leads to a diminished inflammatory response to LPS and decreased expression of PGE2, IL-6, and TNFα (31). Our group has reported that GPER1 is also crucial for E2 anti-inflammatory action in LPS activated primary human monocytes and *in vitro* differentiated human macrophages: GPER1 mediated this effect *via* its direct physical interaction with the 36-kDa ERα splice variant, called ERα36, and the p65 subunit of NFκB. The formation of this hetero-protein complex led to a reduced capacity of NFκB to activate the expression of key molecules like IL-6 and TNFα (32). This is physiologically relevant since we also found expression and co-localization of ERα36 and GPER1 in macrophage cells infiltrating coronary artery atherosclerosis plaques from coronary heart disease patients.

In an elegant set of experiments focusing on liver tumorigenesis, Wei et al. found that GPER1 knockout mice display accelerated hepatocarcinogenesis in the diethylnitrosamine (DEN) hepatocellular carcinoma (HCC) model. This was attributed to increased local inflammation and fibrosis, accompanied by elevated IL-6. Since the major source of IL-6 in the liver are Kupffer cells (liver resident macrophages), they isolated bone marrow mononuclear cells from wild type and knockout mice and found that LPS induced IL-6 production is blocked in these cells in a GPER1-dependent manner (33). These findings are also relevant for human HCC where they found decreased GPER1 expression in tumors versus adjacent non-tumor tissue.

### Eosinophils

Although it has been reported that CD15+ cells do not express GPER1 (19) another group reported GPER1 expression both at the mRNA and protein level in highly purified eosinophils (34). In their study, Tamaki et al. found that G-1 does not provoke eosinophil degranulation or chemotaxis, but increased CCL11-induced chemotaxis. GPER1 effect on eosinophil apoptosis is dependent on their activation status. G1 blocked caspase-3 dependent spontaneous apoptosis of resting eosinophils but had an opposite effect on IL-5 stimulated cells. These findings suggest that low estrogen levels may lead to worsening of eosinophil-dependent conditions *via* loss of GPER1 dependent control. Interestingly, the decline of estrogen levels during the premenstrual period is believed to worsen medical conditions like asthma, a well-known eosinophil-dependent condition (44). Although data in humans are lacking, it was found that GPER1 suppressed airway inflammation in a mouse model of asthma (35). GPER1 could, therefore, be part of the pathophysiology of several eosinophil related diseases that display sexual dimorphism or perimenstrual variation.

### Neutrophils

The expression of GPER1 on the surface of human neutrophils was only recently reported, related to a significantly modified gene expression profile. Previous reports have shown that polymorphonuclear cells express the classical ERα and ERβ estrogen receptors, which were believed to have mostly anti-inflammatory actions (45, 46). Contrary to these reports regarding the effects of estrogen on neutrophils, G1 activation of GPER1 triggered a proinflammatory reaction with increased cytokine (IL1b, CXCL8) and COX2 expression, enhanced respiratory burst and increased life span (36). This finding supports a differential role of GPER1, compared to classical estrogen receptors, in regulating inflammation in these cells. However, it seems that several key pieces of the puzzle are still missing.

Significantly more data regarding the expression and the role of GPER1 in neutrophils come from studies in fish. The research groups involved, however, have reported that in fish G1 has mostly anti-inflammatory effects, *via* changes in the expression profile of acidophilic granulocytes (the equivalent of human neutrophils) (13, 37, 38).

## Mechanisms Related to GPER1 Actions and Interactions With Other Molecules

Most studies on immune-related action of GPER1 have focused on phenotypic events and less is known regarding the underlying signaling mechanisms. In the few studies that included intracellular signaling, the major GPER1-related pathways involved extracellular signal-regulated kinase 1/2 (ERK1/2), phosphoinositide 3-kinase (PI3K), and NFκB (26, 29, 32). In human neutrophils, the major pathways also involved cAMP/protein kinase A/cAMP-response element-binding protein, p38 mitogen-activated protein kinase, and ERK (36).

We have shown that GPER1 physically interacts with ERα36 and the p65 subunit of NFκB. This complex is found both in the cytoplasm and the nucleus, and is related to the estrogen inhibitory NFκB-mediated expression of IL-6 and TNFα (32). Furthermore, other groups have reported functional crosstalk between GPER and other nuclear steroid receptors including the vitamin D receptor (VDR) (47), the glucocorticoid receptor (GR) (48), and the mineralocorticoid receptor (MR) (49, 50) although the latter has been strongly questioned due to lack of proof for aldosterone binding to GPER1 (51). Furthermore, Vivaqua et al. have reported functional and physical interactions between GPR30, activated EGFR and ERα-alpha that may set off complex signaling cascades in hormone-sensitive cancer cells (52). This is an interesting mechanism since in the same study GPER1 was also found to be upregulated by EGF and TGF alpha in endometrial and tamoxifen-resistant breast cancer cells *via* the EGFR/ERK transduction pathway and c-fos (52).

Another controversial finding, related to the effects of steroids *via* GPER1, has to do with the effect of dehydroepiandrosterone (3β-hydroxy-5-androsten-17-one, DHEA), a molecule with a significant functional role in human immunity, to act *via* GPER1 [reviewed in (53)]. It has been reported that rapid DHEA-induced miR-21 transcription involves GPER1, estrogen receptor α-36 (ERα36), EGFR signaling, and activation of c-Src, ERK1/2, and PI3K (54). Although the results of this study have not been followed-up, the interaction of GPER1 with ERα36, also reported by our group, points out that such an interaction might be a more general model of GPER1 action.

## GPER1 Involvement in Immune-Related Human Diseases

As GPER1 is expressed in different human immune cells (presented above) regulating their life span and/or activation, a crucial role of GPER1 in a wide range of immune-related disorders has been suggested. These include chronic inflammatory and autoimmune diseases as well as immunodeficiencies [recently GPER1 deletion has been reported to be central for a case of familial immunodeficiency (55)]. For the scope of this review, we will concentrate on GPER1 involvement in inflammation-associated disorders.

### Neuroinflammatory Disorders

Estrogen was known for many years to be active in the central nervous system (CNS) [see (56, 57) for reviews]. They arrive at their target cells either through the general circulation (by crossing the blood-brain barrier-BBB-) or through local production by neurons or astrocytes (58, 59). Several studies, including ours, reported estrogen to possess antiapoptotic and antioxidant activities [reviewed in (60)], which position them as anti-oxidant and anti-inflammatory agents, in the CNS. These beneficial effects of estrogen have resulted in the investigational use of estrogen in many clinical trials for inflammatory CNS conditions, presented in **Table 2**. The main targets of all these trials were intracellular ERα or ERβ, which are present in astrocytes or glial cells [excellently reviewed in (57)]. However, in our study, before the identification of GPER1 (1, 2), we reported that, in PC12 cells, BSA-bound estrogen mediates anti-apoptotic effects through membrane binding, mobilization of intracellular Ca2+ and activation of specific intracellular kinases pathways, independently from the activation of ERα/β (61). In addition, membrane estrogen binding sites, lately associated with GPER1, were identified in preparations of rat brain tissue. Later on (62), using the same model (PC12 cells), we have reported a detailed intracellular pathway. It includes NOS activation, CREB’s, and NFκB nuclear translocation, leading to a pro-survival effect of estrogen *via* the BCL2-family of anti-apoptotic proteins.

**Table 2:** Clinical trials using estrogen agonists or antagonists in inflammatory CNS conditions.

Condition	Estrogen compounds	
	Agonists	Antagonists
Traumatic Brain Injury	NCT00973674	NCT00065767 (Raloxifene)
Stroke	NCT00026039	NCT00368459 (Raloxifene)
	NCT01040182	
	NCT00005466	
Alzheimer’s Disease	NCT00018343	
	NCT00006399	
	NCT00000176	
	NCT00000177	
	NCT00066157	
	NCT03718494	
	NCT03101085	
	NCT02142777	
	NCT01982578 (Genistein)	
Parkinson’s Disease	NCT00234674	
ALS		NCT02166944 (Tamoxifen)
		NCT01257581 (Tamoxifen)
		NCT00214110 (Tamoxifen)

Source: www.clinicaltrials.gov accessed on May 18, 2020.

The discovery of GPER1 shed a new light on the effect of estrogen in neuro-inflammation. Indeed, GPER1 was found, in addition to neuronal cells (61), also on microglial cells and astrocytes (63–67). Anti-inflammatory effects were attributed to GPER1 in several systems, including cells of the CNS (1, 8, 27, 61, 62, 66–71).

Although no clinical trials are available for the time being, due to the absence of a clinically available specific GPER1 agonist or antagonist, there are compelling preclinical indications about a specific involvement of this receptor in neuro-inflammatory diseases. Indeed, the GPER1 specific agonist G1 was found to be beneficial in an animal model of experimental encephalomyelitis and multiple sclerosis (68), by reducing the severity of the disease and reducing the level of pro-inflammatory cytokines. This effect was also reported by other groups (24, 41) and was attributed to the anti-inflammatory effect of GPER1, mediated by PD1 inhibition (24) [an element which was exploited also in the case of melanoma therapeutic manipulation (72)], or inhibition of pro-inflammatory cytokines (41). G1 also has a protective effect in the 1-methyl-4-phenyl-1,2,3,6-tetrahydropyridine (MPTP) mouse Parkinson’s Disease model. G1 is directly neuroprotective, but most importantly it has an indirect effect through an anti-inflammatory action on immune cells (macrophages, lymphocytes) (69, 73). Finally, GPER1 reduced neural injury and improved neural damage in a mouse model of ischemic brain injury, through inhibition of the TLR4-mediated inflammatory process (66).

### Other Inflammatory Diseases

The anti-inflammatory effect of GPER1 has been investigated in several diseases and conditions, outside the CNS. The main organ that has been investigated is the vascular endothelium. Indeed, many reports (15, 16, 32, 74–77) investigated the anti-inflammatory effects of GPER1-mediated E2 effect in normal and atherosclerotic vessels. In mice with pronounced atherosclerosis, GPER1 deficiency was an aggravating factor, linked to disease progression. The effect of GPER1 was mediated by infiltrating immune cells (macrophages, lymphocytes) and was mediated by the GPER1-induced prostanoid production by the vascular endothelium (78). TNF-induced vascular inflammation (a condition which mimics the cellular stimuli induced by infiltrating immune cells), could also be attenuated by activation of GPER1, and enhanced by GPER1 antagonists, or activation of ERα, suggesting an opposing role of nuclear and extranuclear estrogen actions in the vascular endothelium (79). This finding led the authors to propose specific pharmacological options for GPER1 activation in vascular inflammation and derived atherosclerosis (80) and a specific role of this receptor in the maintenance of heart health (75).

Interestingly, GPER1 seems to play a significant role in large bowel physiology and disease [see (12) for a review]. More specifically, GPER1 seems to be downregulated in Inflammatory Bowel Disease and especially Crohn’s disease, as compared to the normal tissue, suggestive of a protective role of the receptor in bowel inflammation (81). Although the data are not conclusive, the fact that GPER1 is expressed preferentially in normal tissue (81), together with its anti-inflammatory effect on different lineages of circulating or tissue-resident immune cells, as discussed above (28, 31–33, 35, 44), suggest a potential role of this receptor in bowel inflammation, a condition that when is present for prolonged periods of time (chronic colonic inflammation) is a risk factor for colon carcinogenesis (12). For more details on this topic please refer to the specific review in this special issue.

As described previously, GPER1 has been also implicated in liver inflammation, liver fibrosis, and hepatocarcinogenesis (33). In the absence of GPER1 the latter is increased and is accompanied by enhanced immune cell infiltration and production of inflammatory mediators like interleukin-6 (IL-6), through action on stellate cells rather than on hepatocytes, an effect reported previously (82). Therefore, GPER1 may prevent hepatocarcinogenesis *via* its anti-inflammatory effects.

Another condition characterized by a low degree of chronic inflammation is obesity, resulting in the emergence of Type II diabetes (15). In this condition, an underlying low-grade chronic inflammation is considered an important factor leading to insulin resistance. The anti-inflammatory effect of GPER1, documented by the administration of G-1 in experimental animals, verified the importance of this receptor in reducing vascular inflammation in adipose tissue, liver, and pancreas (33, 83–87). Interestingly, another mechanism GPER1 affects diabetes and hypercholesterolemia, is a direct action on lipid metabolism (84) and insulin signaling (84, 85). These effects have a direct impact on the generation and aggravation of type II diabetes, as discussed in detail in another review in the context of this thematic issue.

Finally, by modulating tissue and infiltrating immune cell-regulated inflammation, a role of GPER1 was reported in the regulation of endometriosis (88).

### Cancer and Tissue Micro-Environment Inflammation

GPER1 has also been implicated in cancer and stroma-related inflammation, a hot topic in cancer research, and a preferential therapeutic target in cancer treatment. (The role of GPER1 in cancer is the object of a specific review, in this special issue.)

As discussed above, GPER1 activation inhibits PD1 production and action of pro-inflammatory cytokines, positioning this receptor as an interesting player for the modulation of the tumor microenvironment (24, 41). This element has been exploited in in melanoma (72). Furthermore, GPER1 stimulation by tamoxifen [acting as an agonist on this receptor (2)] inhibits the myofibroblastic differentiation of pancreatic stellate cells in the tumor microenvironment of pancreatic tumors, hampering their ability to remodel the extracellular matrix and to promote cancer cell invasion. GPER1 activation reduces the recruitment and polarization of the M2 phenotype of tumor-associated macrophages, inhibiting tumor inflammation, and immune suppression (87). However, GPER activation by either E2 or G-1 has been found to induce IL1β expression in cancer associated fibroblasts, and IL-1R1 in breast cancer cells, leading to a more aggressive phenotype (89). Furthermore, T-lymphocytes-related apoptosis induction by GPER1 (90, 91), leads to an inability of the major immune cells infiltrating breast stroma, in primary or metastatic breast cancer to support tumor expansion (92, 93). Overall, this positions GPER1 as a good prognostic and/or therapeutic target in several cancers, where the tumor microenvironment is critical for tumor expansion.

In addition to the modulation of the tumor micro-environment, GPER1 activation has a direct immunomodulatory effect on the tumor tissue, *per se*. Indeed, GPER1 was found to be an androgen-repressed gene and is therefore highly expressed in castration-resistant but not in androgen-responsive prostate cancer (94). Through a thorough analysis of xenografted prostate tumors in mice, the authors report that GPER1 up-regulation (and its activation by G1) results in an increased expression of genes related to the interplay between innate and adaptive immunity. Furthermore, they report substantial necrosis of xenografted tumors through increased production of neutrophil attracting cytokines. Therefore, GPER1 is a pro-inflammatory mediator in castration-resistant prostate cancer involved in neutrophil movement, accumulation, adhesion, activation, and phagocytic respiratory burst. Interestingly, a similar E2-induction of the mammary gland with a resulting inflammation was also reported during mammary gland involution (95), although the authors do not specifically investigate the implication of GPER1.

GPER1, through modification of local inflammation and the corresponding immune response, has been reported to play a role in inflammatory breast cancer (74). Specifically, if GPER1 is co-expressed with ERα, it is a good prognostic marker, related to improved overall survival and disease-free survival. GPER1 also increases miR-148a, which in turn induces HLA-G, in both ER+ and triple-negative breast cancer cells (96). The expression of the latter molecule impairs the immune evasion of breast cancer, again suggesting that GPER1 is a good prognostic indicator in breast cancer.

Finally, as discussed above, GPER1 has an indirect impact in colon carcinogenesis through modulation of immune responses (12), while in a thorough investigation, Wei et al. propose that the effect of GPER1 on liver tumorigenesis might be attributed to the anti-inflammatory effect of the agent rather than to a direct action on cancer cells (33).

## Future Perspectives in GPER1 Immunity-Related Research

GPER1 actions on immune functions seem to be abundant and could be critically important, especially in neuro-inflammation and in inflammatory processes related to atherosclerosis. A universal finding across systems and cell types seems to be GPER1 dependent modulation of TLR4 mediated events, with hints that this could be a mechanism affecting several other fundamental pathways exploiting NFκB to lead to inflammation. Current studies have given us just a glimpse of the potential of this molecule and more studies are needed in this area.

The field of GPER1 research has been, however, obscured by “availability bias” characterized by the narrow focus on single molecules and mechanisms. The complexity of estrogen-mediated anti-inflammatory actions may include interactions between GPER1 and classical estrogen receptors or their isoforms, as well as interactions of GPER1 with other nuclear receptors with the capacity to regulate the immune system. The latter is a critical issue for the explanation of the diverse actions of estrogen and G1 on the same mechanism, seen not only in GPER1 action on immune functions but also in other systems. Since the physiologically relevant molecule is estrogen, our current knowledge regarding the effects of G1 suggests that GPER1 could have a more universal role as a central rheostat for diverse intracellular mechanisms related to inflammation. Therefore, future studies on the role of GPER1 on immune functions should focus on a thorough analysis of all the potential molecular interactions and intracellular mechanisms in each cell type and each disease model.

Another important issue that should be further evaluated is the potential role of GPER1 in the mediation of sexual dimorphism in human diseases. It would be interesting to clarify if there exist hormone independent differences in immune cell GPER1 levels between males and females, or if sex-dependent differences in estrogen levels are critical, although both phenomena could be important in different clinical conditions. Sex related differences in the expression of several other GPCRs have already been described and have been related to sex dimorphism in cardiovascular diseases and stress responses (97, 98).

Finally, the site where GPER1 resides is also an interesting research subject. Membrane, endoplasmic reticulum, Golgi apparatus, and nuclear localization of GPER1 (as observed in immune cells), suggest molecular modifications that could also affect its function. Deciphering GPER1 cellular trafficking could also help us find ways to exploit its immune-modulating capacity.

## Conclusions

GPER1 is a fascinating molecule that continuous to surprise us with its diverse functions in the immune system. Since its discovery, it has caused a paradigm shift in the way we understand estrogen actions and the gender-dimorphism of several pathologies. Its role in the immune system only now starts to unravel and initial data are promising. Moreover, the role of GPER1 does not seem to be related only to estrogen. GPER1 seems to have a more universal role in regulating the function of almost all immune cells and several pro-inflammatory mechanisms. Although there are still a lot of uncharted territories to cover, the GPCR nature of GPER1 and the existence of specific agonists and antagonists make it a convenient therapeutic target for the immune system. Hopefully, the best is yet to come.

## Author Contributions

All authors agreed on the initial draft of the review. All authors contributed to the article and approved the submitted version.

## Funding

This manuscript was partially supported by the German Academic Exchange Service (DAAD project-ID: 57515112) and by the University of Crete Special Account of Research Funds (KA10388 and KA10000).

## Conflict of Interest

The authors declare that the research was conducted in the absence of any commercial or financial relationships that could be construed as a potential conflict of interest.
